# Report from the second cytomegalovirus and immunosenescence workshop

**DOI:** 10.1186/1742-4933-8-10

**Published:** 2011-10-28

**Authors:** Mark Wills, Arne Akbar, Mark Beswick, Jos A Bosch, Calogero Caruso, Giuseppina Colonna-Romano, Ambarish Dutta, Claudio Franceschi, Tamas Fulop, Effrossyni Gkrania-Klotsas, Joerg Goronzy, Stephen J Griffiths, Sian M Henson, Dietmar Herndler-Brandstetter, Ann Hill, Florian Kern, Paul Klenerman, Derek Macallan, Richard Macaulay, Andrea B Maier, Gavin Mason, David Melzer, Matthew Morgan, Paul Moss, Janko Nikolich-Zugich, Annette Pachnio, Natalie Riddell, Ryan Roberts, Paolo Sansoni, Delphine Sauce, John Sinclair, Rafael Solana, Jan Strindhall, Piotr Trzonkowski, Rene van Lier, Rosanna Vescovini, George Wang, Rudi Westendorp, Graham Pawelec

**Affiliations:** 1 Department of Medicine, University of Cambridge, Cambridge, UK; 2 Division of Infection and Immunity, University College London, London, UK; 3 School of Cancer Sciences, University of Birmingham, Birmingham, UK; 4 College of Life and Environmental Sciences, University of Birmingham, Birmingham, UK; 5 Mannheim Institute of Public Health, Social and Preventive Medicine, University of Heidelberg, Mannheim, Germany; 6 Department of Pathobiology and Medical and Forensic Biotechnologies, University of Palermo, Palermo, Italy; 7 Epidemiology and Public Health Group, Peninsula Medical School, University of Exeter, Exeter, UK; 8 Dept. Experimental Pathology, University of Bologna, Bologna, Italy; 9 Research Centre on Aging, Immunology Program, Geriatric Division, Faculty of Medicine, University of Sherbrooke, Sherbrooke, Canada; 10 Addenbrooke's Hospital, Cambridge, UK; 11 Stanford University School of Medicine, Stanford, CA, USA; 12 School of Immunity and Infection, University of Birmingham, Birmingham, UK; 13 Dept Microbiology and Immunology, Oregon Health and Science University, Portland, OR, USA; 14 BSMS, University of Sussex Campus, Falmer, UK; 15 NIHR Biomedical Research Centre, Nuffield Dept of Clinical Medicine, University of Oxford, Oxford, UK; 16 Centre for Infection, St George's, University of London, Cranmer Terrace, London, UK; 17 Department of Gerontology and Geriatrics, Leiden University Medical Center, Leiden, The Netherlands; 18 Department of Immunobiology and the Arizona Center on Aging, University of Arizona, Tucson, AZ, USA; 19 Department of Internal Medicine and Biomedical Sciences, University of Parma, Parma, Italy; 20 INSERM, Infections and Immunity, Universite Pierre et Marie Curie-Paris, Paris, France; 21 Instituto Maimonides de Investigación Biomédica de Cordoba (IMIBIC), Hospital Universitario Reina Sofía, Universidad de Cordoba, Cordoba, Spain; 22 Department of Natural Science and Biomedicine, School of Health Sciences, Jönköping University, Jönköping, Sweden; 23 Department of Clinical Immunology and Transplantology, Medical University of Gdańsk, Gdańsk, Poland; 24 Sanquin Blood Supply Foundation and Department of Experimental Immunology, Amsterdam, The Netherlands; 25 Division of Geriatric Medicine and Gerontology, Johns Hopkins University School of Medicine, Baltimore, MD, USA; 26 Department of Public Health and Primary Care, Leiden University Medical Center, Leiden, The Netherlands; 27 Department of Internal Medicine II, University of Tübingen, Tübingen, Germany

## Abstract

The Second International Workshop on CMV & Immunosenescence was held in Cambridge, UK, 2-4^th ^December, 2010. The presentations covered four separate sessions: cytomegalovirus and T cell phenotypes; T cell memory frequency, inflation and immunosenescence; cytomegalovirus in aging, mortality and disease states; and the immunobiology of cytomegalovirus-specific T cells and effects of the virus on vaccination. This commentary summarizes the major findings of these presentations and references subsequently published work from the presenter laboratory where appropriate and draws together major themes that were subsequently discussed along with new areas of interest that were highlighted by this discussion.

## Background

The first International Workshop on CMV & Immunosenescence was held in Tubingen, Germany, December 2009. The discussions focused on trying to define what immunosenescence is exactly, the phenotypes of the cells associated with it and what evidence was available that long term carriage of HCMV into old age was detrimental to health as assessed either by measuring all-cause mortality or degradation of the immune system such that older HCMV-seropositive individuals respond less well to neo-antigens such as seasonal influenza vaccination. The outcome of the first workshop was summarized in this Journal by Pawelec et al. [1]. The attendees at that workshop decided that it would be extremely valuable to meet again in a year's time with a remit to update participants on progress on questions left open from the first meeting's discussion, and to extend participation to other scientists omitted from or unable to attend the first workshop. The Second International Workshop on CMV & Immunosenescence was duly held in Cambridge, UK, 2-4^th ^December, 2010. The second meeting format was modified to include a day of formal seminar presentations with some emphasis on the presentation of data that addressed open questions highlighted in the first meeting. This was then followed by a half-day round-table discussion.

The seminar presentations covered four separate sessions: cytomegalovirus and T cell phenotypes; T cell memory frequency, inflation and immunosenescence; cytomegalovirus in aging, mortality and disease states; and the immunobiology of cytomegalovirus-specific T cells and effects of the virus on vaccination. This commentary summarizes the major findings of these presentations and references subsequently published work from the presenter laboratory where appropriate and draws together major themes that were subsequently discussed along with new areas of interest that were highlighted by this discussion.

## Cytomegalovirus and T cell phenotypes

The impression left on the immune system of the majority of the population (as characterized by changes in T cell phenotype) following primary CMV infection and its subsequent life-long carriage is well-established and uncontroversial. Nonetheless, certain special populations may resist the effects of CMV infection on their immune signatures [2]. As these data stem from observations in exceptionally long-lived families, and because such longevity is likely influenced by multiple genes, sharing of more or less of the latter in the general population is likely to make the impact of CMV a continuous rather than a discrete variable. This complicates interpretation of data treating the presence of CMV infection as simply present or absent (ie. seropositive or seronegative). **Speiser **(Lausanne) presented evidence that the late-differentiated HCMV-specific CD8+ T cell clones in the periphery had a restricted T cell receptor usage and particular clonotypes segregated with particular states of differentiation. Interestingly, once established, these patterns were stable over many years. This may again suggest a subtle genetic control of anti-CMV surveillance.

**Vescovini **and **Sansoni **(Parma) made available their unpublished data on an immune phenotype and frequency study in a cohort of 125 HCMV-seropositive elderly subjects aged between 60-100 years. The study concluded that there was a significant correlation between the magnitude of CMV-specific CD4+ T-cell responses and accumulation of effector T-cells within the CD4+ T cell compartment. There was also a significant correlation in the magnitude of CMV-specific CD8+ T-cell responses and number of total CD8+ T-cells as well as the number of both memory and effector CD8+ T-cells. The study did not find significant correlations between the magnitude of CMV-specific T-cell responses and a shortage of either CD4+ or CD8+ naïve T-cells. In this instance, the presence or absence of CMV infection did seem to behave as a discrete variable in its effects on naïve T cells. These findings emphasize the over-riding impact of CMV infection *per se *on commonly-measured immune parameters, and those commonly attributed to immunosenescence (ie. loss of naïve T cells). **Solana **(Cordóba) presented further evidence that there is an accumulation of CD8+ CD28- T cells with short telomeres that tend to also acquire inhibitory NK receptors in healthy elderly people. He noted that similar changes can be seen in chronic stimulation situations such as patients with melanoma, HIV infection and RA. The HCMV pp65-specific T cells from elderly individuals were found to be heavily enriched in CD45RA+ CD27- CD28- CCR7- cells and evidence was presented that these had a low proliferative capacity following specific stimulation with pp65 peptides [3]. He noted that similar phenotype changes can be seen in HCMV-pp65 specific CD8 T cells in solid organ transplantation that were associated with age and with episodes of CMV replication after transplantation. In addition a correlation was observed between the duration of HCMV replication after transplantation and the percentage of EMRA HCMV-specific CD8+ T cells [3,4]. All these data are consistent with the potential of some (CMV, HIV) but not all (eg. HSV, HCV) [5] chronic viral infections to drive the acquisition of immune signatures associated with "immunosenescence". However, the relationship, if any, between such immune signatures and mortality in any populations except the very old (> 85 yr) Swedes investigated in the OCTO/NONA studies [6] still remain to be established, as does the question of whether the contribution of CMV infection is causative or merely reactive.

**Kern **(Brighton) presented a study to establish the most relevant target proteins recognized by ageing CMV-positive individuals so that a knowledge base can be established to inform researchers which proteins to use for monitoring and analyzing T-cell responses. Currently the focus is very much on pp65 and IE-1; however, many other proteins are recognized by T cells and there is good reason to believe that studies on pp65 and IE-1 alone are not representative of the entire immune response to CMV. The reported study investigates T-cell responses to 19 ORFs (previously identified by Sylwester et al. [7] to be the most important target proteins) in approx. 100 older people (including 50 people over 75+). The study addresses a spectrum of different T-cell functions with respect to each target protein and it will facilitate a wider, more comprehensive view of the T-cell response to CMV in older age. The study will be concluded in 2011 and results are expected to be published in 2012.

## T cell memory frequency, inflation and immunosenescence

Setting aside the difficulties in proving that accumulation of cytomegalovirus-specific T cells with the distinct phenotypic and functional characteristics described above are responsible for an increase in all-cause mortality or a decreased ability to mount good quality responses to new antigens, and whether their specificity is important in this context, both **Hill **(Portland) and **Klenerman **(Oxford) addressed the biological phenomenon of "memory T cell inflation" using the murine CMV model. Klenerman has investigated the mechanism that leads to memory inflation while Hill has asked what happens to the CMV-specific T cell response if cytomegalovirus replication is inhibited. This latter investigation is of particular importance as it has been suggested that elderly HCMV-seropositive people may benefit from anti-herpes virus chemotherapy. The rationale would be that anti-viral therapy would decrease HCMV replication and thus chronic antigenic stimulation which might reduce the frequency of the late-stage differentiated T cell pool and thus allow an increase in the naïve pool with the hope that T cell responses to neo-antigens would improve. **Klenerman **addressed the issue of why memory inflation is triggered by some antigens but not others. Memory inflation is defined as the inordinate increase in frequency of CD8+ T cells specific for certain antigens with time post-exposure to antigenic stimulation. The phenomenon has been seen with CMV, HIV and parvovirus B19 infections; however, certain antigens from these viruses but not to others within the same virus are found to cause this effect, and other viruses do not cause it at all. The "inflating" cells have a distinct transcriptional profile as compared to non-inflating T cells from the same animal. It is not clear if persistence of viral antigen is required regardless of viral reactivation or why some epitopes cause inflation while others do not. The Klenerman study concluded that viral replication was not required for memory inflation and that epitopes that showed inflation had a reduced dependence on the immunoproteasome [8]. **Hill **also addressed memory inflation in MCMV specifically to understand what happens to the T cell response if cytomegalovirus replication was inhibited? Using two separate models of replication and spread-deficient virus that still establishes viral latency, memory T cell inflation nonetheless occurred and was maintained. Inhibition of viral replication by inhibiting viral DNA replication also had no effect on the T cell response over time [9].

Taking these two studies together, the data from the murine model would suggest that treating humans with anti-herpesvirus chemotherapy may not reverse the effects of HCMV on the memory T cell compartment.

Most studies of this type have been carried out in humans or mice, but **Nikolich-Zugich **(Tucson) presented data looking at the maintenance of T cell memory in the face of CMV and ageing using the Rhesus CMV (RhCMV) model. The study examined the RhCMV CD4+ and CD8+ T cell responses in adult animals aged 7-10 years and aged animals 19-26 years old. The study concluded that there was no difference in the phenotype, numbers, proliferative capacity or functionality of the T cells between the two groups of animals and that there was no evidence for either a functional decline or exhaustion in aged animals [10]. This was further corroborated by their study using systemic HSV-1 infection, which closely models MCMV infection in mice [11]. However, given that CMV and HSV have very different effects in humans [5], this result in monkeys is perhaps not unexpected.

Further, in the context of the impact of HCMV on immune signatures, **Sauce **(Paris) presented her studies on the impact of HCMV infection in young adults thymectomized during early childhood. The data presented suggested that HCMV infection tended to cause the exhaustion of the naïve T cell pool [12]. Moreover, in a separate study in HAART-treated HIV patients, mounting strong CMV-specific responses appeared to impact on both naïve CD4+ T-cell counts and their recovery upon antiretroviral therapy [13]. In these two clinical settings, T cell responses to HCMV have a profound impact of the immune system as a whole. In the special cases of thymectomy and HIV infection, the impact of CMV may therefore be more pronounced and easier to determine unequivocally.

Further discussion considering the impact of CMV on immunosenescence led to revisiting the question of *what is the definition of T cell senescence? *Senescence is a term generally used to describe replication incompetence of fibroblast cells after a certain finite number of population doublings. Replicative senescent cells have been defined in the brain and in cardiology but how should it be done for the immune system? Are there any such cells in the constantly-renewing T cell compartment? The use of the term in describing CMV-specific T cell populations is more controversial as it is acknowledged that many of the late-differentiated T cells do retain replication competence. However, it should be noted that their definition by CD45RA expression and lack of either CD27 or CD28 or both does not define a homogeneous cell population, as many more markers can be added (eg CD57, KLRG1, PD1) to further sub-divide late-stage T cell populations which may yet include an antigen-specific population that is indeed unable to divide further. Another complication is that the term "senescent" is also being used to imply non-functionality, whereas data from many laboratories in human, rhesus and murine systems have shown that late-differentiated cells maintain function. In vivo, the work of **Macallan **(London) suggests that these cells turn over only very slowly. There was a feeling that we might have become too fixated on the replication capacity of these cells. Of more importance could be their direct effector function. Indeed, the suggestion was made (**Pawelec**, Tübingen) that some of the defining characteristics of senescent cells which T cells share with the "classic" fibroblast model (ie. lack of proliferative capacity, apoptosis resistance, inflammatory mediator secretion) are not markers of senescence in the sense that they are detrimental (which by definition of the term "senescence" has to be shown to justify its use). Instead, one could view these properties as cellular protection mechanisms responsible for maintaining an active population of cells required for immunosurveillance, ie. required for prevention of peripheral clonal deletion of necessary cells. In fact, there is some evidence from the longitudinal NONA studies that at the terminal phase of life in the very elderly, the number of CD8+ clonal T cell expansions, most of which are specific for CMV, is inversely related to remaining survival time [14]. More recently, data from a 7-year follow-up of subjects 88 years old at baseline in the Leiden 85+ study has shown that the degree of accumulation of late-differentiated CD8 cells, putatively mostly specific for CMV, is positively associated with longer survival (Derhovanessian et al., 2011 submitted).

### Discussion of CMV and Immunosenescence - Is it associated at all?

If chronic challenge with CMV contributes to causing immunosenescence, it might be expected that more frequent CMV reactivation would accelerate this process. However, it is not known whether CMV does reactivate more frequently in the elderly. It was felt that higher HCMV IgG levels might reflect this, but in many sick people they stay the same, so did this mean that HCMV was not reactivating? A study of elderly people suffering from acute bacterial infections (e.g. S. pneumonia, E. coli, S. aureus, K. pneumonia) did not show any reactivation of CMV infections measured by IgM antibodies (n = 20). However, serology does not measure viral levels so this is a mute point. Nonetheless, it was strongly felt by a number of clinicians involved with the care of older adults that *"It is very unlikely that CMV reactivation and viral replication is a problem in the very elderly." ***Wang **(Baltimore) provided some evidence for this, indicating that a small-scale study of older adults (n = 40) categorised as either frail or not frail showed no HCMV DNA in serum in either group, with the exception of one frail individual who had 8 copies/mL (this would be on the very edge of detection). People were reminded that VZV reactivation occurs in the elderly and that it had been correlated with decrease in CD4 cells and decrease in IFN gamma responses [15]. Data on CMV are lacking.

## HCMV in aging, mortality and disease states

In the studies of Swedish octogenarians and nonagenarians (OCTO and NONA) an immune risk profile (IRP) associated with increased mortality on 2, 4 and 6-year follow-up was defined, comprising reduced B cell numbers, an inverted CD4:CD8 T cell ratio, increased frequency of CD8+ CD28- T cell populations, a poor proliferative response to mitogen stimulation and HCMV seropositivity. **Strindhall **(Jönköping) outlined the results from a new study examining whether this immune profile is already established in a cohort of much younger older people in their sixties, the HEXA study. The initial results show that this profile does exist in hexagenarians. Almost 15% of the cohort had an inverted CD4:CD8 ratio, a frequency very similar to that seen in 85-year-olds, and these individuals also showed characteristics previously identified in IRP individuals of the very old. The study now has the opportunity to examine morbidity and mortality to determine if this truly defines a risk profile in people only in their sixties.

It is clearly of great importance to establish if long-term carriage of HCMV is associated with an increased likelihood of all-cause mortality. A number of studies addressing this issue have already been published [16,17] and the results from further studies were presented at this meeting. **Wang **spoke about a study of 635 70-79 year-old women living in the community. The results demonstrated that women in the highest HCMV IgG quartile had a significantly higher incidence of frailty as compared to HCMV-seronegative women at baseline. Furthermore, CMV IgG concentration in the highest quartile also increased the risk of five-year mortality, independent of potential confounders including cardiovascular disease [18]. Work on data taken from the National Health and Nutrition Examination study (NHANES) III concluded that CMV-seropositivity was associated with an increased risk of all-cause mortality in > 14,000 adults at least 25 years of age followed for > 10 years; especially individuals with a raised inflammatory marker (C-reactive protein CRP) were at a higher risk of both all-cause mortality and cardiovascular-associated mortality [19].

It should be noted that not all studies do show such an association, and that when they do, this does not necessarily imply causality. **Maier **(Leiden) presented results from four cohorts comprising the Leiden 85-plus study, Danish Twin study, Leiden Longevity Study and the PROSPER study with a mortality follow-up of 6 to 12 years. The results did not support a relationship with CMV serostatus or antibody levels in all-cause mortality or inflammation markers in any of the 4 cohorts. Consistent with this, a recent study presented at the 2011 Cytomegalovirus Workshop in Nuremberg, Germany by Stuart Adler concerned a prospective longitudinal study with an 18 year follow-up of 915 patients undergoing coronary angiography. It was concluded that neither CMV seropositivity nor antibody levels were associated with longevity irrespective of the presence or absence of coronary heart disease (personal communication). These data are therefore different from a smaller, shorter, study from Finland on one-third the number of patients with stable cardiovascular disease which did show a positive relationship between highest quartile IgG antibody titer and mortality [16]. This crucial question therefore remains to be resolved, as discussed intensely at the Workshop.

### Discussion on Cohort studies of all-cause mortality CMV seropositive vs negative

It was felt that defining the confounding factors and how to correct for them is crucial in these analyses, although it is not clear what best practice is. The dissimilar populations in these cohort studies likely also contribute to the divergent findings on the association between HCMV carriage and mortality. However, the requirement for standardization in the quantitation of HCMV IgG levels would also be an important step and was discussed further along with other metrics that require standardization. These are outlined in the new questions section of this report.

### Discussions on the Immune risk profile, IRP

The IRP was defined in the Swedish OCTO and NONO studies, but it was left as an open question if the IRP actually applies generally to human populations? It was suggested that a cohort in Spain does show the IRP (**Solana**) and **Moss **has cohorts in the UK which also show the IRP; however, not all cohorts do, as an aged community in the mountain region in Sicily do not show the IRP (**Caruso**, Palermo). However it was felt that this population was not old enough as so far we only know that the IRP is relevant from age 85.

The IRP does not take into account the absolute number of naïve T cells, nor the absolute numbers of memory cells: the IRP was based on percentages. The percentage of CD8+CD28- cells was part of the IRP, but it is also clear that there is an increase in the absolute number of these cells per ml of blood in the risk group. This raises the question at what levels of naïve CD4 or CD8 T cells do people start to experience problems? **Sauce **suggested from her work that a level of < 50 naive CD8+ T cells per μl and < 100 naïve CD4+ T cells is crucial, although different specific cut-offs might be relevant at different age ranges. A further refinement of the accumulation of CD8+ CD28- T cells defined in the IRP was suggested to take into account lymphopenia, as it was felt that those individuals who were both lymphopenic and had large CD8+ CD28- expansions had a poor outcome.

## The immunobiology of cytomegalovirus-specific T cells and effects on vaccination

**Macallan **presented a study to determine why highly differentiated HCMV-specific CD8+ T cells accumulate and in particular to distinguish accumulation due to rapid proliferation versus long-term persistence due to impaired apoptosis. They demonstrated that these expansions are often oligoclonal and these clones are stable over many years. In addition, comparison of CD8 cells from young and old donors showed no difference in either proliferative activity or cytotoxic function. Assessment of in vivo turnover rate by deuterated glucose experiments suggest that the cells have a low proliferative rate in vivo and as such probably accumulate with time rather than by proliferation [20].

**Henson **(London) reported that late-stage memory cells also express inhibitory receptors such as PD-1 and KLRG1 and this tends to be higher on T cells from individuals who are HCMV-seropositive. This has been observed by many others, including several Workshop participants and seems to be a general finding. She showed that blockade of PD-1 increased proliferation of these cells and no additive effect was seen with dual PD-1 and KLRG-1 blockade, nor did it increase telomerase activity.

The idea that CD8+ memory T cells compete for space in a T cell compartment that is constrained in its maximum size has led to the suggestion that over-abundant memory T cells such as those seen in people with latent HCMV carriage could have a detrimental effect by displacing naïve or other memory T cells. Could the occupation of the "immunological space" by large and inflating HCMV-specific T cells contribute to immunosenesence? **Van Lier **(Amsterdam) presented data from a study comparing the frequency of EBV- and HCMV-specific T cells in the blood and lymph node (LN) of donors showing that while the percentages of EBV-specific T cells were comparable between blood and LN, HCMV had a much lower LN frequency which included the late-differentiated cells as determined by CD45RA+ and CD27- phenotyping. The study concluded that strong immune responses to HCMV were unlikely to restrict space for naïve or other memory T cells in LN. Although these results are intriguing, the impact of aging was not addressed in that study. In particular, as CMV infection has been shown to impair the response to a co-resident EBV infection in middle-aged and elderly persons, but not in young adults [21], the study raises a very important question. If CMV infection does not impair the survival of naive T cells in the LN, the major reservoir of naive T cells, how is the decline of naive T cells in the peripheral blood of CMV-seropositive versus CMV-seronegative persons explained? [22,23]. The ability of CMV to significantly decrease peripheral naive T cells in young persons with potentially intact or at least more residual thymic T cell generation indicates that CMV infection may not only drive peripheral naive T cell exhaustion but also affect T cell generation in the thymus. Accordingly, there is a need to delineate the mechanism of naive T cell generation/exhaustion and CMV infection as well as to analyze the impact of CMV infection not only in the peripheral blood, but also in lymphoid and extra-lymphoid organs, to improve our understanding of HCMV-specific T cell migration, differentiation and expansion throughout life.

**Westendorp **(Leiden) presented a study on the efficacy of influenza vaccination in a cohort of elderly individuals living in care facilities. The study design encompassed a number of different dosing regimes and was able to examine influenza seroconversion in relation to HCMV seropositivity. The study concluded that in this particular cohort previous CMV infection does not explain poor responsiveness to influenza vaccination and as such does not support CMV infection as being the origin of this dysfunction in the immune system of these older people [24]. These results contrast with an earlier study [25], in which elderly HCMV-seropositive individuals where shown to respond less well to influenza vaccination. It should be noted that only the dominant H3N2 strain of influenza strain was used in the den Elzen study as opposed to all three seasonal strains in the Trzonkowski study, and that the weakest correlation was between HCMV seropositivity and seroconversion to H3N2 in the latter. It is clear that this important area deserves further study. If HCMV does drive immunosenescence, a tangible outcome such as poor vaccination responses would be predicted and importantly this evidence could support a rationale for a modified vaccination protocol in elderly HCMV seropositive individuals.

## New Questions raised by the discussions

### Standardization - HCMV serotesting the means of detection, the measurement of viremia and T cell assays?

It is not at all clear what antibody responses are actually being measured when HCMV serotesting is reported. There is a requirement for standardization in the CMV antigens that are used on the plate coatings. This is of particular importance as studies of the effect of HCMV on mortality or measures of health often group patients based on the level of antibody response (into four quartile groups). Comparison of different studies is not helped if what is being measured in one study/laboratory is not the same as another study/laboratory. It was felt that there is a need for standardization such that results can be compared, and for further sophistication in dissecting out antibody repertoires. The subclass of IgG is never considered; IgM titer rarely, the avidity of antibody never. All these factors could be informative.

In a similar fashion, concerns about the comparability of T cell assays between groups in the field was felt to be an issue. Very little standardization was evident between different flow cytometry-based T cell assays. Parameters that were of particular concern were: the use of fresh vs frozen PBMC samples, the anti-coagulants used to collect blood samples, the combination of cell surface phenotypes used to determine T cell subpopulations (variations on the use of CD27 or CD28 with CD45RA eg). These concerns are not unique to this field. **Kern **noted that there was a move to adopt a standardization provision of minimal information on T cell assays (MIATA). Similar concerns were raised from a series of conferences on the measurement of antigen-specific immune responses (MASIR). A goal for presentation at the next meeting is to determine a set of recommendations for standardization along the lines outlined in [26,27].

Where are the CMV-specific T cells, where is the reservoir, is it the spleen? Or perhaps marginal pools? This reservoir, and there may be more than one, seems dynamic and provides rapid access to the blood, as peripheral blood cell numbers show rapid fluctuations. The stress hormone adrenaline can regulate this exchange with the blood. **Bosch **(Birmingham) presented data showing that physiological stressors (exercise, psychological stress) cause swift (< 10 minutes) and robust (up to +300%) release of differentiated (CD27-CD28-CCR7-) memory CD8+ T cells as well as CMV-specific CD8+ T cells into peripheral blood. Using pharmacological infusion and blockade studies, he provided evidence that this release is regulated by the stress hormone adrenaline and dependent on activation of β-adrenergic receptors. This finding coincides with reports of upregulated expression of β2-adrenergic receptors on differentiated T cells [28]. Significantly, 1 to 2 hours post-stress these cells cleared from the blood, with cell numbers dropping to 70% below resting levels [29,30]. Bosch suggested that this may reflect a rapid egress into tissue.

In accordance with their effector-memory phenotype, CMV-specific T cells should preferentially migrate to and reside in extra-lymphoid organs and the bone marrow. Yet the frequency of CMV-specific CD8^+ ^and CD4^+ ^T cells in the human bone marrow is similar [31,32] or even lower compared to the peripheral blood [33]. Nonetheless, Letch et al. did show that CMV-specific CD8 cells with a central memory phenotype, while present in low frequencies in the peripheral blood, were present at higher levels in the BM. Moreover, CMV-specific T cells from the BM underwent greater clonal expansion in vitro than PB cells. This might also be of practical importance in transplantation (independently of any ageing considerations). Although the impact of aging on the frequency of CMV-specific T cells in the bone marrow has not yet been addressed, the currently available data may support a role of the bone marrow as a reservoir for highly functional CMV-specific central-memory T cells (**Herndler-Brandstetter**, Birmingham).

### Are large expansions of HCMV specific T cells detrimental?

**Moss **asked us to directly address the issue that if CMV has any association with immunosenescence, old people without CMV must have better immune responses to neo-antigens. It was further suggested that CMV-seropositive vs negative showed no correlation with inflammatory markers - and it would be expected that it should. However, could there be a subset or group of certain individuals that are affected and this is not seen at a population level? **Moss **suggested this was the case in a cohort of CML patients where those with large CD4+ CD28- T cell expansions with a loss of naïve cells showed an increase in infections.

### Is it how someone deals with CMV over a long period of time which is actually important?

The Leiden Longevity Study examines familial longevity in an F2 generation with a 30% decreased standardised mortality rate between the ages of 40 and 80. This is proposed to be due to a genetic influence - an enrichment of "longevity-assurance" genes. The F2 subjects studied here showed some indications of resistance to becoming infected with CMV even when their partner (sometimes over decades) was CMV-seropositive. Moreover, when they were infected, they did not show the characteristically lower frequencies of naïve CD8+ T cells and higher frequencies of late-differentiated CD8+ CD28- T cells seen in the general population [2]. They also showed lower levels of markers of inflammation in the plasma and less potentially inflammatory-mediated disease (eg. type 2 diabetes). The link between CMV, inflammation, disease, longevity and genetics is being studied in these remarkable people.

### Would treatment of CMV with antivirals have a beneficial effect on the immune system?

Evidence from **Hill**'s studies suggest not! However, is the MCMV model sufficiently close to HCMV that we can extrapolate? In the face of the not yet sufficiently documented (and to some extent controversial) health risks of persistent CMV infection, it is highly speculative whether long-term treatment with antiviral drugs will result in any measurable health benefits. In particular, long-term treatment with antiviral drugs may not be a good option, as the treatment is likely to have side effects and to facilitate viral resistance. Some people felt that this argued in favour of the ongoing efforts to develop an efficient CMV vaccine able to protect from primary infection. Such a vaccine would most likely be cost-effective in preventing from any detrimental CMV-related impairments of the immune system.

It was felt that currently-available antivirals also have too much toxicity and adverse-effect burden to tip the benefit-risk balance toward warranting administration to otherwise healthy humans. The administration of these antivirals to immunosuppressed transplant recipients is warranted because of the significant morbidity and mortality caused by CMV in this patient population. If further evidence suggests that halting HCMV replication and reactivation confers a beneficial effect on the immune system, the development of next-generation antivirals with significantly less toxicity would be a high priority.

## Conclusions

This 2^nd ^Workshop was attended by twice the number of people than the first original discussion group. The interaction of so many additional scientists with expertise in basic cytomegalovirus immunology, virology and epidemiology was invaluable. As a group, a large number of the most important questions are being addressed and importantly new questions are being raised and discussed which is vital for the field to move forwards. It was proposed that a 3^rd ^meeting should be organised and this had overwhelming support. We are grateful to Rafael Solana (rsolana@uco.es) for offering to do this in Cordoba, Spain, 8-9^th ^March, 2012.

## Conflict of interests

The authors declare that they have no competing interests.

## Authors' contributions

All authors contributed to the writing of this Commentary and all have approved the final version.
